# Slight pelvic obliquity is normal in a healthy population: a cross-sectional study

**DOI:** 10.1186/s40634-023-00613-z

**Published:** 2023-05-31

**Authors:** Alireza Moharrami, Peyman Mirghaderi, Nima Hoseini Zare, Mir Mansour Moazen-Jamshidi, Mazaher Ebrahimian, Seyed Mohammad Javad Mortazavi

**Affiliations:** 1 Joint Reconstruction Research Center, Tehran University of Medical Sciences, Tehran, Iran; 2 Surgical Research Society (SRS), Students’ Scientific Research Center, Tehran University of Medical Sciences, Tehran, Iran; 3 Fellowship of Hip and Pelvic Surgery, Zanjan University of Medical Science, Zanjan, Iran; 4 Orthopedic Resident, Tehran University of Medical Science, Sina Hospital, Tehran, Iran

**Keywords:** Normal range, Pelvic obliquity angle, Pelvis, Reference values

## Abstract

**Purpose:**

Pelvic obliquity (PO) has not been extensively investigated, and there is no gold standard for measurement. The PO is essential for surgeons in planning hip arthroplasty, which includes the restoration of leg length discrepancy (LLD). We aimed to establish a normative range of PO angles by measuring healthy individuals without musculoskeletal disorders.

**Methods:**

Our study included 134 consecutive cases (70 females) referred to our institution between April 2020 and September 2021 for non-orthopedic problems. Patients were screened for normal gait and posture using the visual observation method (VOM) and the Modified Gait Abnormality Rating Scale (GARS-M). In standing standard radiographs, the PO angle was measured as the angle between the horizontal plane and the inter-teardrop line.

**Results:**

Patients' mean age and Body Mass Index (BMI) were 39.7 ± 16.8 and 22.3 ± 3.1, respectively. PO angles did not follow a normal distribution, with a median (IQR) of 2.0° (0.9°—3.1°). According to the Wilcoxon one-sample test, the median PO angle differed significantly from zero (*P* < 0.001). The PO angle did not differ significantly between males and females (2° vs. 2°, *P* = 0.46), nor did it correlate significantly with age (*P* = 0.24). Considering the 95% percentile of PO angles was 5.6°, this range (0°—5.6°) was regarded as a normative value.

**Conclusion:**

Normative values for PO in the normal healthy population range from 0° to 5.6°, with a median value of 2.0°. The PO angle was independent of age and sex and differed significantly from 0°. Slight pelvic obliquity may be normal, and physicians should not always assume that it is caused by pain, scoliosis, or weakness of the abductors.

**Level of evidence:**

III.

**Supplementary Information:**

The online version contains supplementary material available at 10.1186/s40634-023-00613-z.

## Introduction

Pelvic obligity (PO) has not been extensively studied, and there is no gold standard for measuring it [8]. A balanced body weight on each hip side is the only way to achieve an upright position. A significant PO disturbs this harmonic balance, resulting in secondary lumbar scoliosis, accelerated spinal degeneration, and abnormal gait patterns [1]. PO could originate from suprapelvic, intrapelvic, and infrapelvic etiologies [6]. Distortion of the spinal column results in supra-PO, and alteration of the pelvic bones results in intra-PO. Hip muscle contracture results in infra-PO whether the hip is adducted or abducted [19]. Thus, detecting PO may facilitate diagnosis of underlying conditions. Moreover, PO is essential when planning hip arthroplasty, including cup implantation and restoring leg length discrepancy (LLD) [6, 10]. Coronal pelvic malrotation causes the bony acetabulum to be displaced from its normal location. In patients with PO greater than 6 degrees, cup inclination may increase, which could lead to cup instability [19]. It may be necessary to rebalance the pelvis through preoperative skeletal traction or release hip contractures to address this problem [10].

PO can be defined as a deformity between the spine and the pelvic axis [3]. Several methods for measuring PO offer good to excellent inter- and intra-rater reliability, but there is no agreement on the best method. Using the line of eburnation and the intersulcate line, Fann et al. present two methods for measuring PO [4]. Zhou et al. describe a slightly different way of measuring PO by using the iliac crest and fourth lumbar vertebra and classifying them into six groups [19]. Similarly, Torretti et al. utilized the inferior parts of the sacroiliac joint and the anterior superior iliac spines [15].

PO can result from various etiologies, including spinal deformity, hip contracture, leg length discrepancy (LLD), antalgic positions, muscle weakness, and idiopathic reasons [17]. The previous literature has not reported a range for PO in healthy adults. PO ranges were reported in some studies but not as normative values. In 2020, Otayek et al*.* reported that the mean PO angle in asymptomatic patients is – 0.1 ± 1.6 [11]. For scoliosis patients, Banno et al. defined significant PO as > 3°, but did not have anatomical references for the ranges they used [2, 20].

The present study aimed to define a normative range of PO angles by measuring healthy people without musculoskeletal disorders.

## Materials and methods

**Fig. 1:**
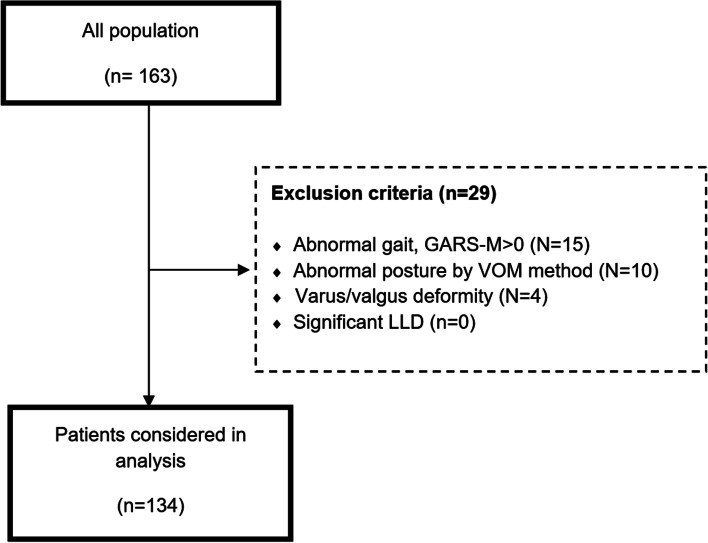
Patient enrolment flowchart

**Fig. 2:**
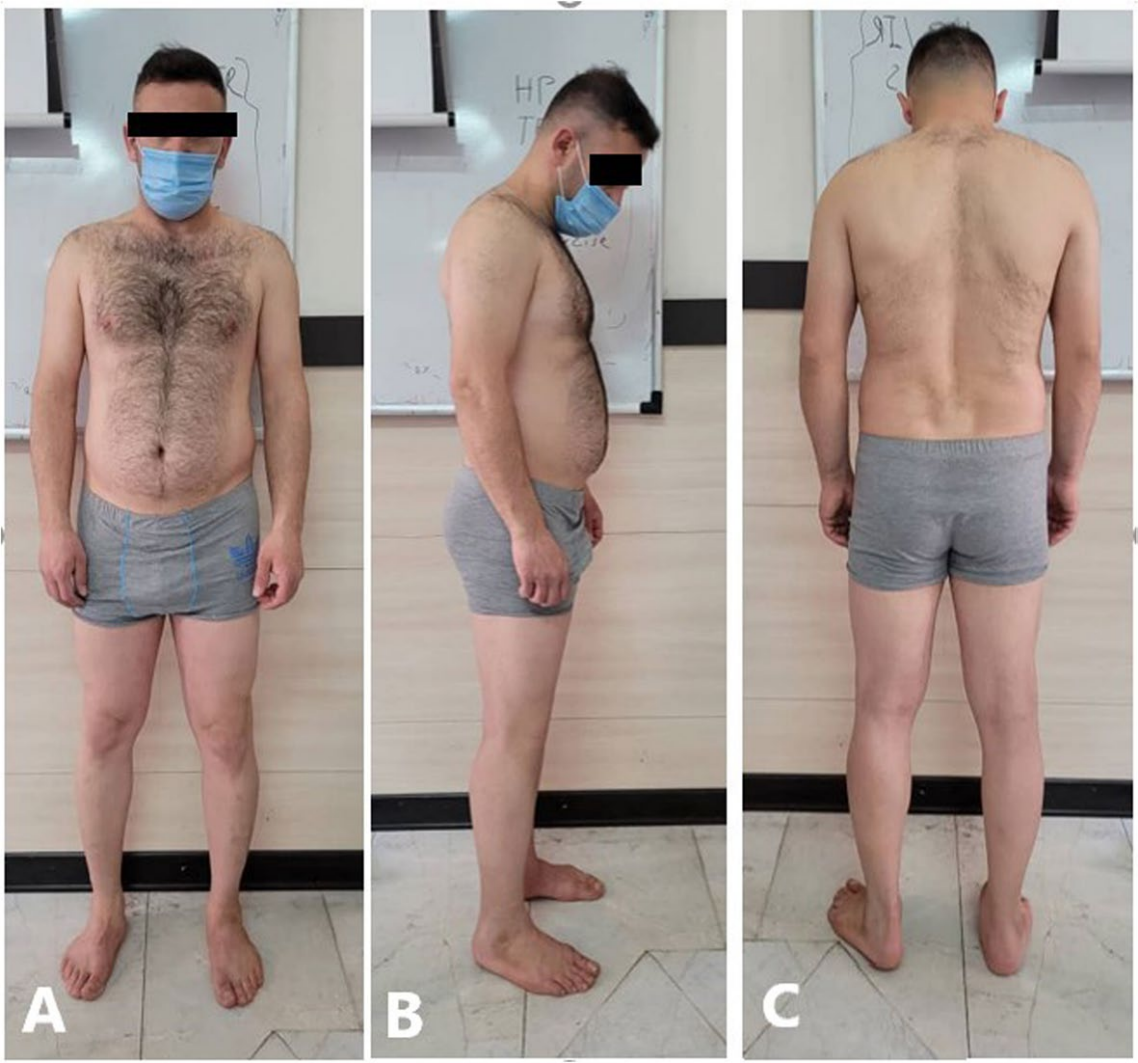
A patient standing upright during a screening for posture and gait in a clinic **A** Anterior (frontal plane), **B** lateral (sagittal plane), **C** Posterior (screening lordosis kyphosis scoliosis)

**Fig. 3:**
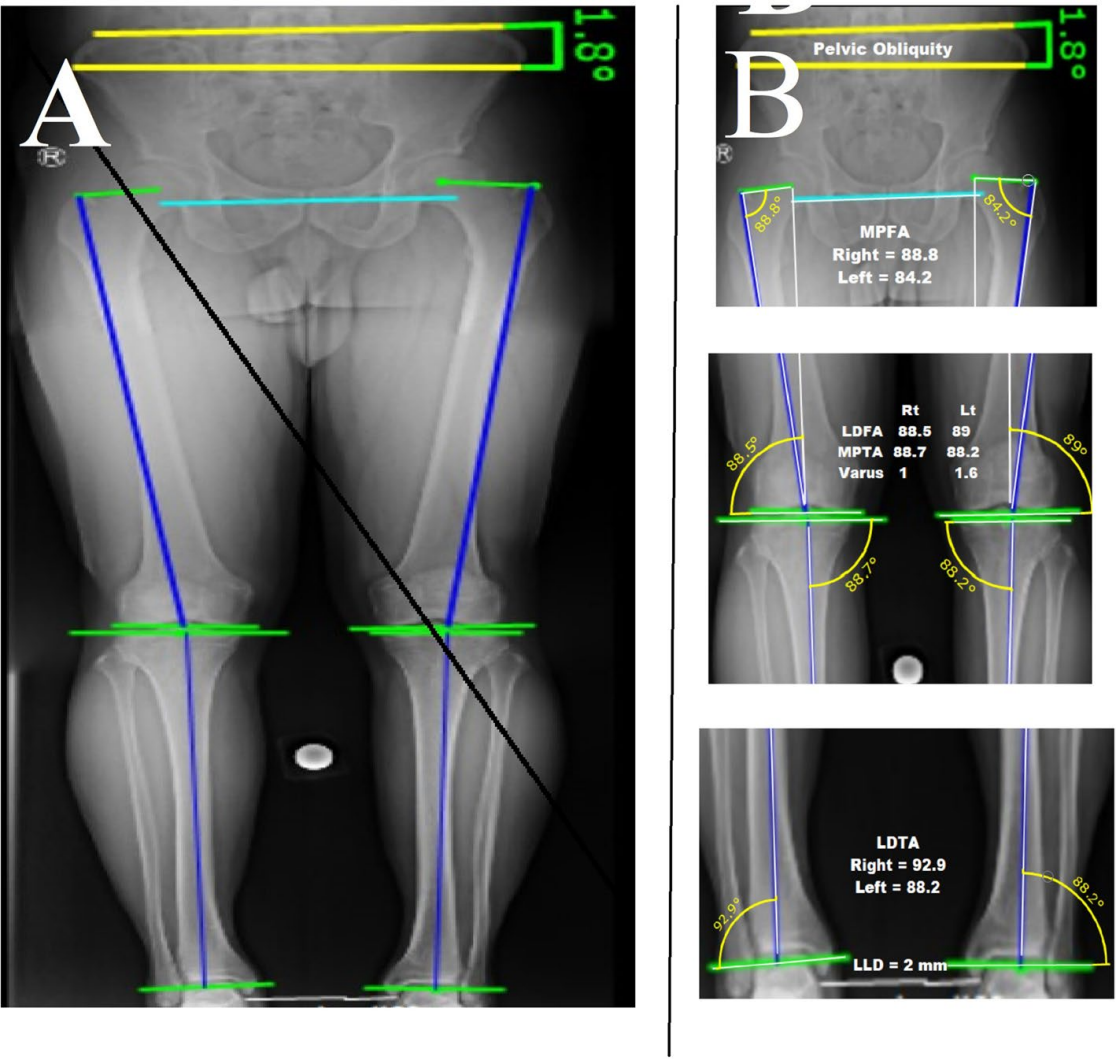
**A** A standard three-joint view, the blue line is the inter-teardrop line, **B** from a zoomed view. Significant LLD (> 1 cm), and significant varus/valgus lower limb mal-alignment (using MAD) are considered exclusion criteria that are measured in the 3-joint x-ray

We conducted a cross-sectional study of 134 consecutive cases (70 females and 64 males) referred to our institution for non-orthopedic problems from April 2020 to September 2021. Inclusion criteria included patients without spinal, pelvic, or lower extremity abnormalities or a history of orthopedic surgery. Any gait or posture abnormality, significant LLD (> 1 cm), and significant varus/valgus lower limb mal-alignment were considered exclusion criteria (Fig. 1). We evaluated the patient's posture and gait abnormality with the Modified Gait Abnormality Rating Scale (GARS-M) [18]. The patients were scored from 0 (No abnormality) to 21 (severe) based on the seven parts of the questionnaire (Supplementary Table 1). GARS-M inter-rater reliability coefficients were greater than 0.90 [16]. Patients with a GARS-M score of zero were included (*N* = 15 excluded). Also, the postural state was assessed by the visual observation method (VOM) [12, 13]. After head-to-toe examination, this method classified the patient's posture into 14 types (P1 to P14), including head, neck, shoulder, thorax, trunk, lordosis, kyphosis, scoliosis, hip, knee, and foot postures (Supplementary Table 2) (Fig. 2). Therefore, we excluded patients with any posture abnormality from the VOM method (*N* = 10 excluded). Further, all patients were evaluated by standard 3-joint views (Fig. 3), and their LLD and varus/valgus angles were measured. For measuring varus and valgus, we used mechanical axis deviation (MAD) in the 3-joint x-ray (Fig. 3). Significant malalignments revealed by MAD >  + 1 SD or MAD < -1 SD were excluded. Before radiography, patients were requested to consent and did not have to pay, as the imaging was carried out exclusively for this study.

**Fig. 4:**
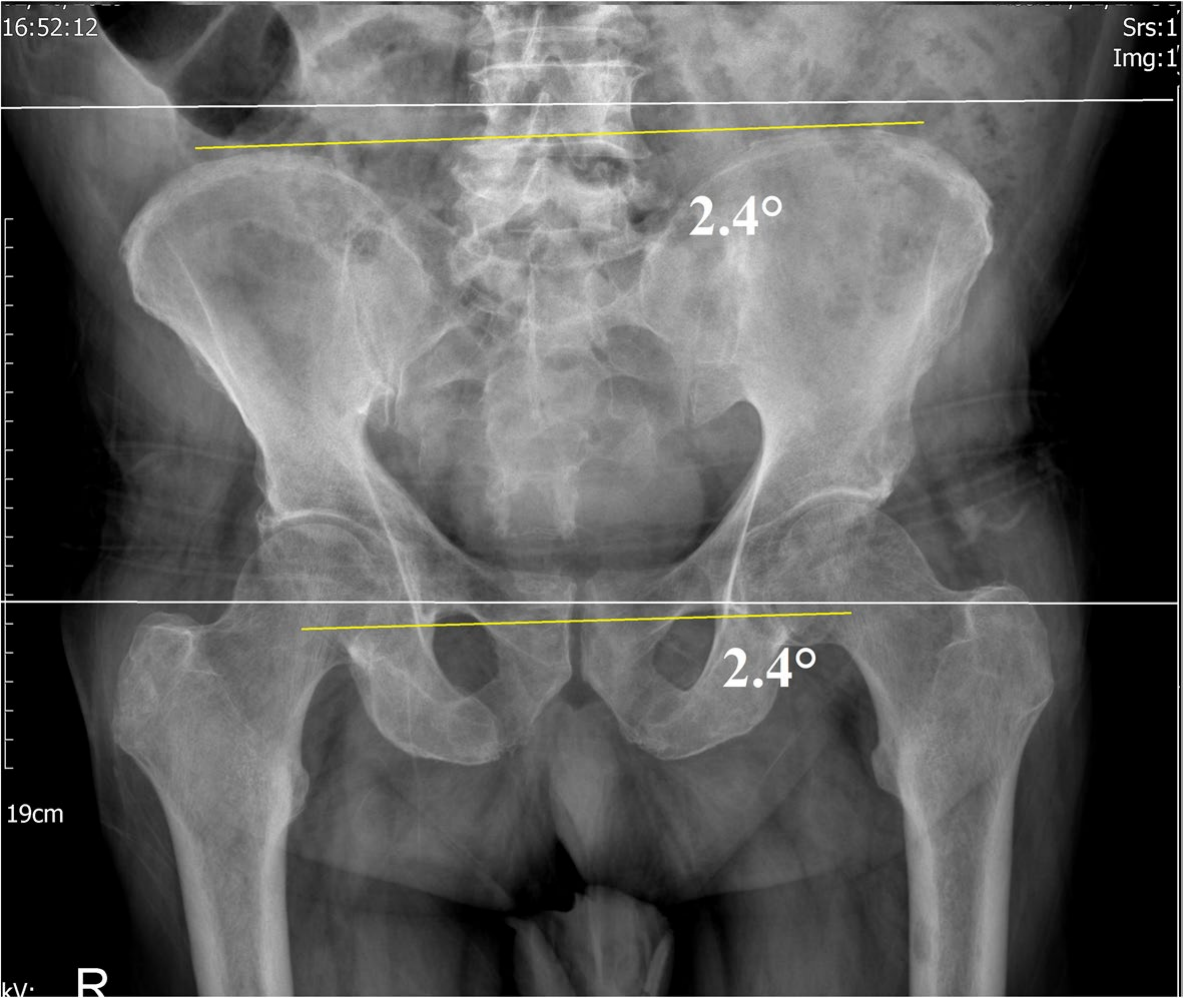
Pelvic obliquity (PO) angle measurement in AP standard pelvic radiography. A PO angle is the angle between the horizontal plane (white lines) and the inter-teardrop line (inferior yellow line, the upper yellow line is parallel)

For AP X-rays of the pelvis, all patients underwent the same protocol in a similar radiology department. The patients were standing, both legs in 15° internal rotation. Film and tube distances were standardized at 1.15 m. The beam was centered on the pubis symphysis. The coccyx should be aligned with the pubic symphysis with 2.5 cm intervals between them in the female sex and 1.5 cm in the male sex, as described by Tannast et al. [14]. PO angle was measured as the angle between the horizontal plane and the inter-teardrop line (Fig. 4) [5] using mediCAD® software (version 3.5, Hectec, Landstuhl, Germany) [9]. Heidt et al. compared the variations of the O'Brien method and revealed that the angle formed by the horizontal reference line and the inter-teardrop line was most reliable for measuring pelvic obliquity [7].

Finally, data were analyzed with IBM SPSS software (version 25.0, USA). Data are presented as mean and standard deviation (SD) for normal continuous variables and median, interquartile range (IQR) for non-normal continuous variables, while categorical data are presented as numbers and percentages. Kurtosis, skewness, Kolmogorov–Smirnov, and Shapiro–Wilk tests were used to determine whether the samples had a normal distribution. One-sample Wilcoxon test was used to compare the PO angle and zero. Mann–Whitney U and Spearman's correlation tests were used to analyze the association between PO angle and sex and age. *P*-values of less than 0.05 were considered significant.

The Institutional Review Board (IRB) reviewed and approved the study design and confirmed no ethical concerns in the present study. Furthermore, we obtained informed consent from all patients in this study.

## Result

There was a mean age of 39.7 ± 16.8 and a mean Body Mass Index (BMI) of 22.3 ± 3.1. Kurtosis and skewness tests indicated that PO angles were not normally distributed (kurtosis and skewness to standard error ratio = -0.32 and 3.4). The Kolmogorov–Smirnov and Shapiro–Wilk tests were also in agreement (*P* < 0.05).

**Fig. 5:**
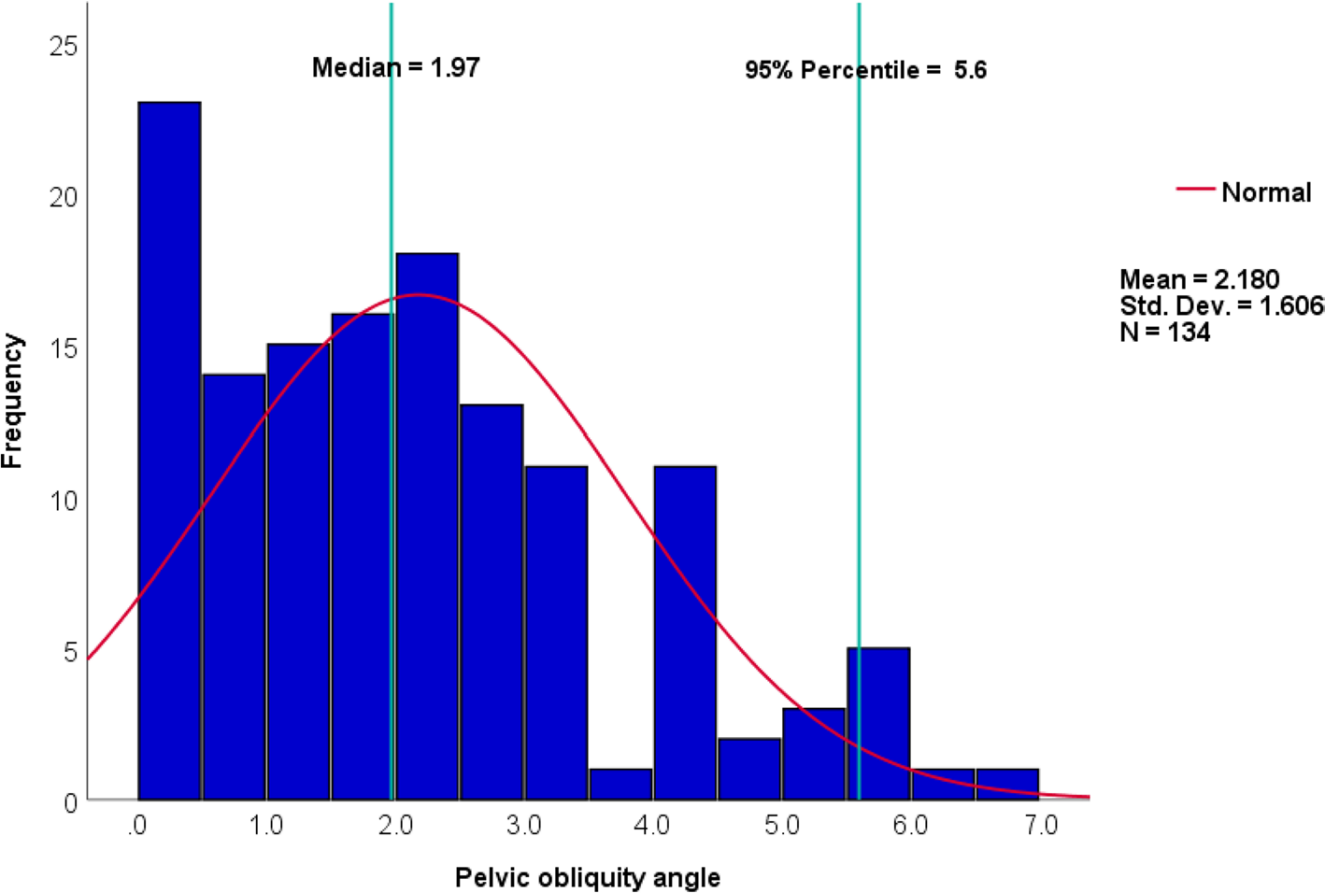
The non-normal distribution of pelvic obliquity angle, median, and 95% percentile

**Fig. 6:**
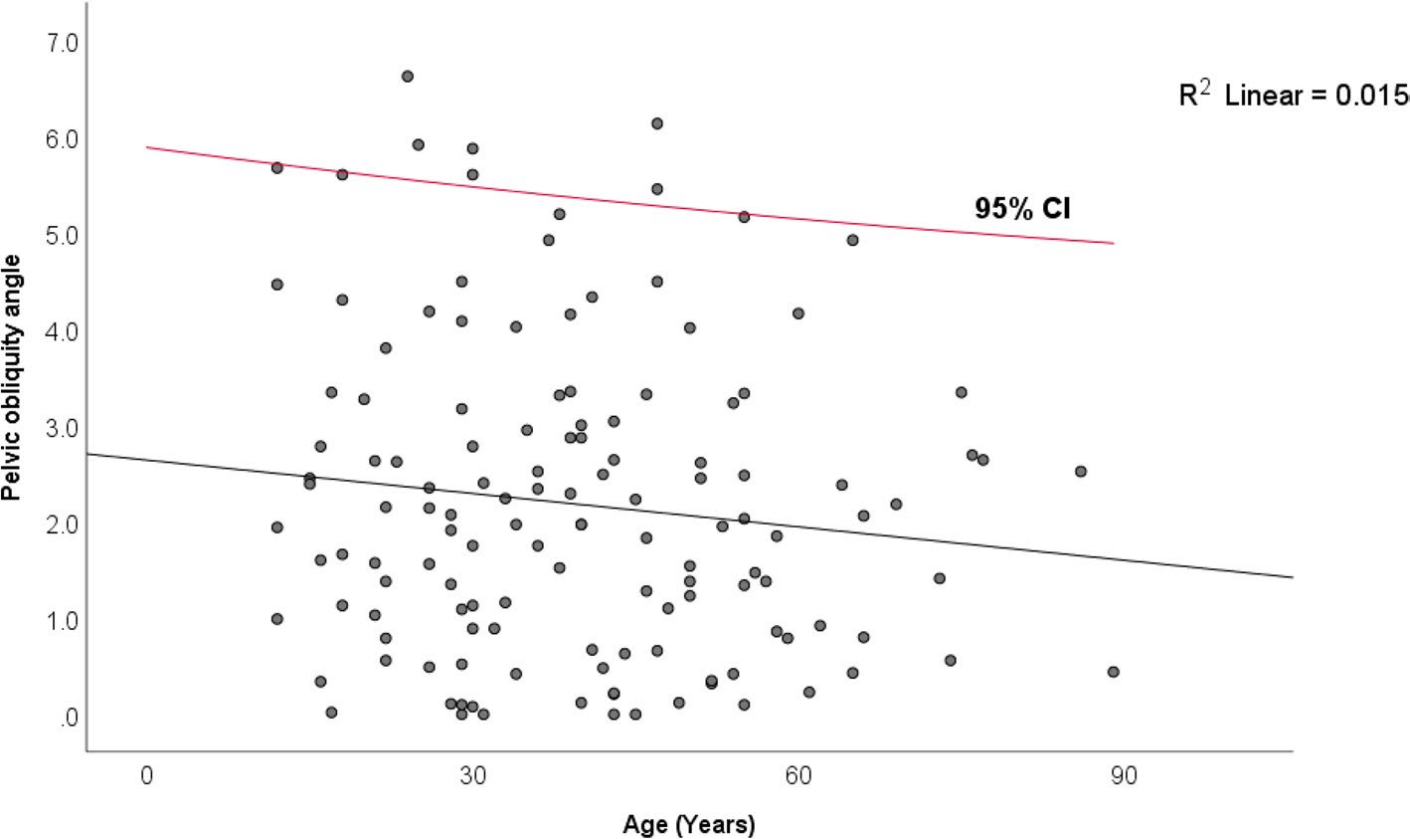
No significant correlation between PO angle and age (*P* = 0.24)

The median (IQR) PO angle was 2.0° (0.9 – 3.1) (Fig. 5) (Table 1). The 95% percentile of PO angle was 5.6°. The median PO angle was statistically different from zero based on the one-sample Wilcoxon test (*P* < 0.001). According to the Mann–Whitney U test, there were no significant differences in PO angle between males and females (2.0° (0.7 – 2.8) vs. 2.0° (0.9 – 3.3), *P* = 0.46). There is no correlation between age and PO angle (Spearman correlation coefficient = -0.10, *P* = 0.24) (Fig. 6) (Table 1).

**Table 1:** Pelvic obliquity (PO) angle in Normal Population

	Median (IQR)	Mean (SD)	Minimum	Maximum	*P*
Total (*N* = 134)	2.0 (0.9 – 3.1)	2.2 (1.6)	0	6.6	< 0.001^2^
Sex					
Male (*N* = 64)	2.0 (0.7 – 2.8)	2.1 (1.6)	0	6.4	0.46^1^
Female (*N* = 70)	2.0 (0.9 – 3.3)	2.3 (1.6)	0	6.8	

^1^Mann–Whitney U test

^2^One-sample Wilcoxon test (Comparing the median PO angle with zero)

## Discussion

This study found that the median (IQR) of PO angle in the normal healthy population without musculoskeletal problems was 2.0 (0.9—3.1), significantly different from zero (*P* < 0.001). PO angles of 95% of the patients were less than 5.6°, suggesting that 0°—5.6° is the normal range for PO in a healthy normal population (Fig. 5). In addition, PO angle was not associated with age and sex (*P* > 0.05).

Studies have used various measurement methods to determine PO, so a precise definition and measurement method remains unclear. PO measurement is essential for surgeons planning hip arthroplasty procedures, including placing a cup and restoring LLD [6, 10]. Coronal pelvic malrotation leads to an abnormal position of the acetabulum. A PO greater than 6 degrees was associated with increased cup inclination, leading to cup instability [19]. In some cases, this requires rebalancing the pelvis through preoperative skeletal traction or the release of hip contractures [19]. Also, THA may affect intra-PO, the most common cause of LLD. PO before THA is a crucial factor in post-operative PO [10].

According to our knowledge, previous literature mainly addressed the measurement of PO and the importance of PO changes in patients with various problems. An issue that was not addressed and discussed clearly was the normal range of PO angles or the use of zero degrees as a norm. Otayek et al. in 2020 reported the mean PO angle in their study as – 0.1 ± 1.6, and they declared that the patients were asymptomatic [11]. Still, they did not define the normative range for PO angle based on the radiological and clinical examination. Banno et al. considered PO ≥ 3° as a definition of PO in patients with scoliosis, but they had no anatomical references for the considered range [2]. Our study defined a new normal range of PO for further studies, unlike other research that used zero degrees as a reference point. Thus, having a slight obliquity can be normal, and surgeons should not always attribute it to other factors such as pain, abductors' weakness, scoliosis, etc.

In this study, PO was measured as the angle between the horizontal plane and inter-teardrop line or between the acetabular teardrops described by Hagglund et al. [5]. There are several methods to describe PO angle. A review study by Dubousset et al. discusses PO simply as a deformity discovered among the spine and oblique axis; however, the emphasis on the axis must be considered because the spine and pelvis are three-dimensional structures. They also defined PO in three ways that can be explained briefly: 1. a plastic bone that may be inaccurate with growing, 2. intercalary bone between the trunk and lower limbs 3. one individual vertebra that can be displayed in three dimensions [3]. Fann et al. [4] showed PO angle with three lines. Line A was the line drawn parallel to the line of eburnation or the intersulcate line. Line B was drawn parallel to the plumb line through the apex of each femoral head. Line C was perpendicular to the plumb line. The height between line B and the point where line A and line C meet is the PO angle. Zhou et al. [19] revealed a simple method, and Torretti et al. [15], discussed it. Two lines described their method: one line linked the top of both iliac crests on AP pelvis radiography, and the second was the lowest part of the fourth lumbar vertebra, and PO angle defined where two lines intersect. This method couldn't be used when the lower lumbar spine is not clear. In this situation, the angle between the inter-teardrop line and horizontal lines is the best alternative method for measuring PO angle. Zhou et al. also categorized the angle that was calculated into six groups with a variation of three degrees which started with zero degrees and were defined as A and B. Their definition depends on the side of PO, which deviates to the right or left.

Some causes of PO include muscle contracture related to the hip joint, not similar length of legs, spine deformity and pelvis disorders, or a combination of these reasons. If we notice PO, we must examine the hip and spine and perform both radiography to understand the reason(s), which have different treatments such as fixing the spine deformity and balancing both legs.

There were limitations to this study. First, we did not assess PO angle with other measurement methods but only by evaluating the angle between the horizontal plane and the Inter-teardrop line. The second limitation was that we only studied the normal PO angle in the small sample size, which could limit generalizations to the entire population. Moreover, the cohort consisted of patients without musculoskeletal problems; however, these were not healthy adults as they were referred to a clinic for non-orthopedic problems. Thus, healthy asymptomatic volunteers should be recruited to identify PO in the completely healthy normal population.

## Conclusion

The normal range of PO angle was 0° to 5.6° degrees, with 95% of the population having a PO angle within this range. A physiologic PO angle was not associated with age and sex and differed significantly from 0.

## Supplementary Information

**Additional file 1: Supplementary Table 1.**Modified Gait Abnormality Rating Scale (GARS-M) described by Wolfson et al in 1990. **Supplementary** **Table 2.**The postural examination from head-to-toe with visual observation method (VOM).
