# Supplementary material for: Slight pelvic obliquity is normal in a healthy population: a cross-sectional study
Source: J Exp Orthop. 2023 May 31;10:57. doi: 10.1186/s40634-023-00613-z (PMC10229507; doi:10.1186/s40634-023-00613-z)
Supplement: Supplementary file 1 — Additional file 1: Supplementary Table 1.Modified Gait Abnormality Rating Scale (GARS-M) described by Wolfson et al in 1990. Supplementary Table 2.The postural examination from head-to-toe with visual observation method (VOM). [file 40634_2023_613_MOESM1_ESM.doc]

| **Supplementary Table 1.** Modified Gait Abnormality Rating Scale (GARS-M) described by Wolfson et al in 1990 |
| --- |
| 1. Variability-a measure of inconsistency and arrhythmicity of stepping and/or arm movement 0 = fluid and predictably paced limb movements 1 = occasional interruptions (changes in speed) approximately 25% of the time 2 = unpredictability of rhythm approximately 25%-75% of the time 3 = random timing of limb movements |
| 2. Guardedness-hesitancy, slowness, diminished propulsion, and lack of commitment in stepping and arm swing 0 = good forward momentum and lack of apprehension in propulsion 1 = center of gravity of head, arms, and trunk (HAT) projects only slightly in front of push-off, but still good arm-leg coordination 2 = HAT held over anterior aspect of foot and some moderate loss of smooth reciprocation 3 = HAT held over rear aspect of stance phase foot and great tentativeness in stepping |
| 3. Staggering-sudden and unexpected laterally directed partial losses of balance 0 = no losses of balance to side 1 = a single lurch to side 2 = two lurches to side 3 = three or more lurches to side |
| 4. Foot contact-the degree to which heel strikes the ground before the forefoot 0 = very obvious angle of impact of heel on ground 1 = barely visible contact of heel before forefoot 2 = entire foot lands flat on ground 3 = anterior aspect of foot strikes ground before heel |
| 5. Hip ROM-the degree of loss of hip range of motion seen during a gait cycle 0 = obvious angulation of thigh backward during double support (10") 1 = just barely visible angulation backward from vertical 2 = thigh in line with vertical projection from ground 3 = thigh angled forward from vertical at maximum posterior excursion |
| 6. Shoulder extension-a measure of the decrease of shoulder range of motion 0 = clearly seen movement of upper arm anterior (15") and posterior (20") to vertical axis of trunk 1 = shoulder flexes slightly anterior to vertical axis 2 = shoulder comes only to vertical axis or slightly posterior to it during flexion 3 = shoulder stays well behind vertical axis during entire excursion |
| 7. Arm-heel-strike synchrony-the extent to which the contralateral movements of an arm and leg are out of phase 0 = good temporal conjunction of arm and contralateral leg at apex of shoulder and hip excursions all the time 1 = arm and leg slightly out of phase 25% of the time 2 = arm and leg moderately out of phase 25%-50% of the time 3 = little or no temporal coherence of arm and leg |
| Minimum Score = 0 (No abnormality in the Gait)  Maximum Score = 21 (Severe Abnormality in the Gait) |

| **Supplementary Table 2.** The postural examination from head-to-toe with visual observation method (VOM) | | | |
| --- | --- | --- | --- |
| **P** | **Detail** | **Posture** | **Patients with abnormality (excluded)** |
| **P-1** | Head position in the frontal plane | Head posture | 0 |
| **P-2** | Neck position in the frontal plane | Neck posture | 0 |
| **P-3** | Shoulder position in the frontal plane | Posture of the shoulders | 0 |
| **P-4** | Shoulder position in the sagittal plane | Posture of the shoulders | 0 |
| **P-5** | Thorax aspect | Thorax posture | 0 |
| **P-6** | Shoulder blades position | Posture of the shoulder blades | 0 |
| **P-7** | Scapulae alatae | Posture of the shoulder blades | 0 |
| **P-8** | Lordosis | Trunk posture | 3 |
| **P-9** | Kyphosis | Trunk posture | 2 |
| **P-10** | Scoliosis | Trunk posture | 0 |
| **P-11** | The triangle formed by the lateral part of the trunk, and the medial part of the forearm and upper arm, established in the upright position (the Lorenz’s triangle). In case of scoliotic poor posture, the Lorenz’s triangle is greater on the concave side. | Trunk posture | 0 |
| **P-12** | Spina iliac anterior superior position | Posture of the hips | 0 |
| **P-13** | Knees aspect | Posture of the knees | 0 |
| **P-14** | Flat feet | Posture of the feet | 5 |
